# Chrono-Nanomedicine at the Barrier: Circadian Rhythms Influence the Delivery of Donepezil Across the BCSFB

**DOI:** 10.3390/ijms27156644

**Published:** 2026-07-25

**Authors:** Maria Rodrigues Cardoso, Ana Catarina Duarte, Rafael Mineiro, Miguel Ferreira, Ângela Sousa, Eugenia Gallardo, Hiroshi Ishikawa, Christian Schwerk, Horst Schroten, Cecília Santos, Diana Costa, Telma Quintela

**Affiliations:** 1 RISE-Health, Department of Medical Sciences, Faculty of Health Sciences, University of Beira Interior, Av. Infante D. Henrique, 6200-506 Covilhã, Portugal; 2 Pharmacology and Toxicology Laboiratory, UBIMedical, University of Beira Interior, 6200-000 Covilhã, Portugal; 3 Problem Group Related to Substance Dependence, Beiras Academic Clinical Center (CACB), 6200-000 Covilhã, Portugal; 4 Laboratory of Clinical Regenerative Medicine, Department of Neurosurgery, Faculty of Medicine, University of Tsukuba, Tsukuba 305-8575, Japan; 5 Pediatric Infectious Diseases, Department of Pediatrics, Medical Faculty Mannheim, Heidelberg University, 69117 Mannheim, Germany; 6 UDI-IPG—Unit for yhe Development of the Interior, Polytechnic University of Guarda, 6300-559 Guarda, Portugal

**Keywords:** blood–cerebrospinal fluid barrier, chitosan, circadian rhythms, nanotechnology

## Abstract

To reach the target tissue within the central nervous system (CNS), drugs, such as donepezil (DNPZ), must overcome naturally occurring barriers. Despite its substantial pharmacological relevance, the blood–cerebrospinal fluid barrier (BCSFB) remains insufficiently studied. The BCSFB harbors a functional molecular clock that regulates, for instance, the expression of membrane transporters. Thus, there has been an increasing interest in exploring chronotherapeutic strategies to enhance cerebral drug delivery. Additionally, nanotechnology constitutes a well-established approach for improving tissue-specific drug bioavailability within the CNS through, for example, the employment of chitosan (CS)-based nanosystems. Given the considerable potential of both approaches for the treatment of neurological disorders, we propose establishing an integrated chronotherapy–nanotechnology platform to optimize pharmacological regimens. In this context, this study aims to explore the influence of circadian rhythms in the transport of free and encapsulated DNPZ forms across an in vitro model of the BCSFB. We developed and characterized CS-based nanoparticles with promising properties for the enhanced delivery of DNPZ across brain barriers. We found that free and encapsulated drug forms of DNPZ display distinct patterns of circadian trafficking across BCSFB. In summary, our findings represent an important step toward the integration of chronotherapy and nanotechnology as a promising strategy to optimize therapeutic outcomes.

## 1. Introduction

Alzheimer’s disease (AD) constitutes the primary cause of dementia worldwide [1]. This neurodegenerative condition is clinically characterized by an array of cognitive dysfunctions, including progressive memory loss and spatiotemporal disorientation [2,3]. Associated with a highly complex etiology, AD can be familial or sporadic, with the latter accounting for the vast majority of diagnoses [1,2,4]. At the molecular level, AD is characterized by the deposition of amyloid beta plaques and the accumulation of intraneuronal tangles of phosphorylated tau protein [1,5]. While disease-modifying therapies have gained well-deserved attention in recent years [6], first-line treatments remain focused on symptom management, including N-methyl-D-aspartate receptor antagonists (e.g., memantine) and cholinesterase inhibitors [7,8,9]. The latter includes, for instance, donepezil (DNPZ), a reversible inhibitor proven to enhance cognitive function in mild to severe cases of AD [10]. Despite its established therapeutic efficacy, DNPZ is associated with poor cerebral bioavailability, partly due to the limiting effects of brain barrier systems. Brain barriers, the designated guardians of the central nervous system (CNS), maintain brain homeostasis and include structures such as the blood–brain barrier and the blood–cerebrospinal fluid barrier (BCSFB). The BCSFB is composed of the ciliated epithelium of the choroid plexus (CP), resting upon a layer of loose connective tissue and a complex network of fenestrated capillaries [11,12]. Besides functioning as a physical and biochemical barrier, the CP is a region of bioactive compound synthesis and secretion, with the cerebrospinal fluid (CSF) as the structure’s main secretory product [11,12]. The pharmacological relevance of the BCSFB lies in its active role in regulating blood-to-CSF and CSF-to-blood compound trafficking. To such exchanges, contribute the membrane-transporting systems expressed in the BCSFB, including the solute carrier (SLC) and ATP-binding cassette (ABC)-type transporters [13,14,15].

Nanotechnology emerges as a strategy in which the production of specialized delivery systems increases the cerebral bioavailability of diagnostic and therapeutic compounds, thereby surpassing biological barriers [16,17]. Due to the inherited versatility, biocompatibility, and biodegradability, chitosan (CS)-based nanoparticles (NPs) are amongst the most commonly used polymeric nanosystems [18,19]. CS-based and CS-coated NPs have been explored to enhance the biological performance of drugs used to treat AD, with very promising results [20,21,22].

More recently, chronotherapy, an approach that explores the timed administration of drugs in accordance with an individual’s biological rhythms, has gained considerable attention in the field of personalized therapy [23,24]. Circadian rhythms (i.e., free-running endogenous rhythms with approximately 24-h periods) are crucial regulators of numerous physiological, metabolic, and behavioural processes in nearly all living organisms [25,26,27]. Although generated by intrinsically autonomous machinery, endogenous rhythms are synchronized with the external environment via a range of environmental cues (e.g., light, temperature, food intake), commonly known as *zeitgebers* [26,28]. In mammals, the circadian system operates as a hierarchy, with a master clock in the suprachiasmatic nucleus of the hypothalamus synchronising secondary and tertiary structures [29,30,31]. The presence of functional machinery driving cellular clockwork has been described in numerous tissues, including the CP [32]. Within the barrier, circadian rhythms influence CSF secretion and the expression of components of adherens junctions and membrane transporters, namely SLC and ABC-type systems [33,34,35]. Thus, considering a chronotherapeutic approach, exploring BCSFB-associated rhythms may allow an increase in the cerebral bioavailability of numerous drugs.

Given the recognized potential of nanotechnology and chronotherapy in the treatment of neurological disorders, we propose integrating these strategies to optimize existing pharmacological regimens. In this context, we explored the influence of circadian rhythms in the transport of free and encapsulated DNPZ forms across an in vitro model of the BCSFB. Specifically, to the best of our knowledge, this study demonstrates for the first time that free and encapsulated DNPZ exhibit distinct circadian-dependent transport profiles across the BCSFB.

## 2. Results

### 2.1. Circadian Oscillations of BMAL1 and ABCG2 mRNA Levels in HIBCPP Cell Line

**Figure 1:**
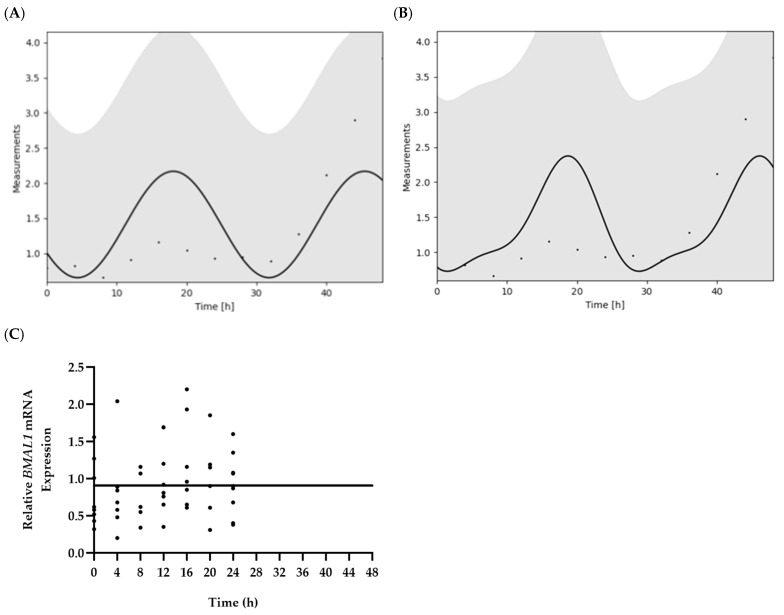
Circadian transcription profile of *BMAL1* in HIBCPP cells. (**A**) Differential rhythmicity analysis performed with Multi-Component cosinor regression. (**B**) Differential rhythmicity analyses performed with Single-Component cosinor regression. (**C**) Data graphics following CircWave analysis. The yielded slope of zero demonstrates no significant rhythmic variation in the data (*p* > 0.05).

The circadian expression of the core clock gene *BMAL1* was assessed to characterize circadian rhythmicity in an immortalized human epithelial CP (HIBCPP) cell line. Significant rhythmicity was obtained with JTK_CYCLE analysis (*p* = 0.030) (Table 1) and the Multi-Component cosinor model with a user-defined 24-h period (*p* = 0.041) (Figure 1A; Table 2). Using JTK_CYCLE, the main rhythm-associated parameters for *BMAL1* expression were estimated as a period of 28 h, a phase of 18 h, and an amplitude of 0.19 (Table 1). When the 48-h *BMAL1* expression data were analyzed using Multi-Component regression with a user-defined 24-h period, the fitted parameters included a period of 27.38 h, a midline estimating statistic of rhythm (MESOR) of 2.22, an amplitude of 1.56, and acrophase and center of gravity (CoG) values of zero. In contrast, the Single-Component (Figure 1B) and Multi-Component cosinor models fitted with a user-defined 27 h period failed to identify a significant rhythm, as indicated by *p*-values of 0.1154 and 0.107, respectively (Table 2). Oscillations in *BMAL1* mRNA levels were not considered statistically rhythmic when subjected to CircWave analysis (*p* > 0.05) (Figure 1C).

**Figure 2:**
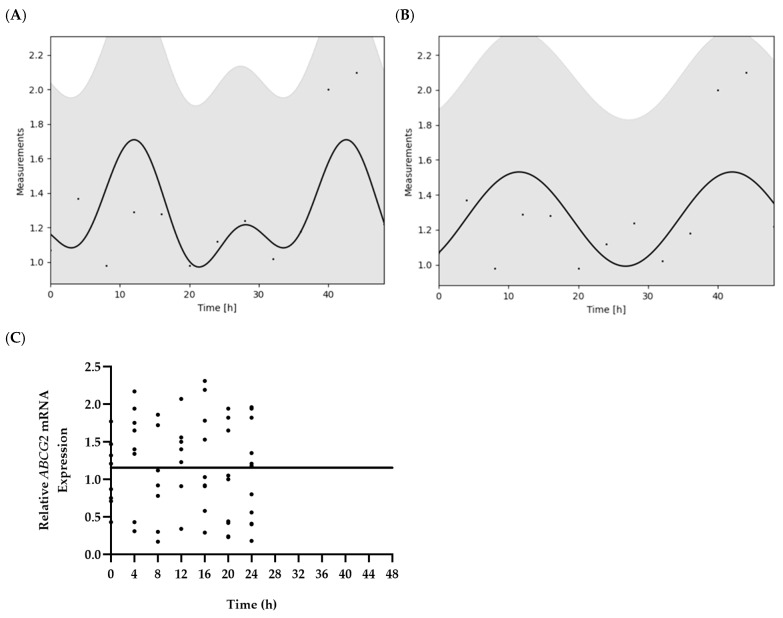
Circadian transcription profile of *ABCG2* in HIBCPP cells. (**A**) Differential rhythmicity analysis performed with Single-Component cosinor regression. (**B**) Differential rhythmicity analyses performed with Multi-Component cosinor regression. (**C**) Data graphics following CircWave analysis. The yielded slope of zero demonstrates no significant rhythmic variation in the data (*p* > 0.05).

Considering the expression data obtained for *ABCG2*, significant rhythmicity was detected with the Single- (Figure 2A; Table 2) and the Multi-Component cosinor model with a user-defined period of 27 h (Figure 2B; Table 2), as analysis using both Single- and Multi-Component regression models resulted in an estimated period of 30.53 h, a MESOR of 1.54, and an amplitude of 0.56. The Single-Component model estimated an acrophase of −2.77 and a CoG value of −13.47 h. In comparison, the Multi-Component model showed an acrophase of −2.93 and a CoG value of −14.25 h. No significant rhythm was obtained with JTK_CYCLE analysis (Table 1), Multi-Component cosinor regression with a user-defined period of 24 h (Table 2), and CircWave analysis (Figure 2C).

### 2.2. Development of DNPZ-Loaded Chitosan Nanoparticles

**Figure 3:**
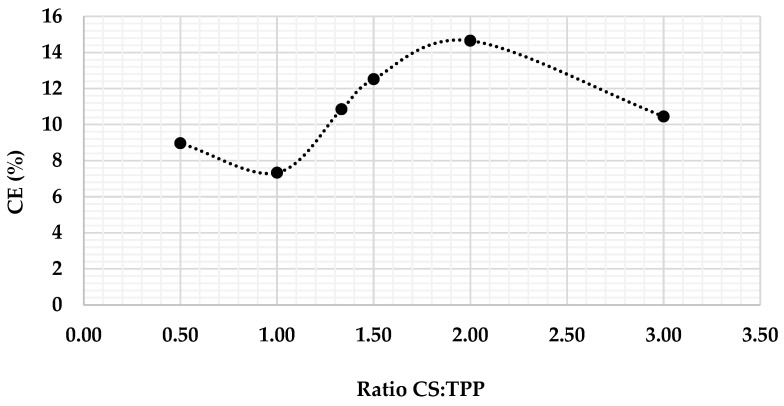
CE (%) values plotted as a function of the respective CS:TPP ratio. A maximum peak in CE was obtained for the 2:1 ratio.

CS-based NPs loaded with DNPZ were synthesised by ionotropic gelation at varying CS: tripolyphosphate (TPP) ratios, as previously described [22]. Values of process yield and complexation efficiencies (CE) of synthesis reactions attending to each CS:TPP ratio are summarized in Table 3. CE values were plotted as a function of the respective ratio (Figure 3).

The 2:1 and 3:1 ratio were selected for further characterization based on the best combined yield and CE values for each formulation. Despite the association with a more adequate combination of yield and CE values, in formulations with a 4:3 ratio, visible aggregates formed immediately after synthesis. Measured average sizes for 2:1 and 3:1 NPs were 90.02 ± 2.152 nm and 98.47 ± 2.094 nm, respectively. Moreover, obtained polydispersity index (PDI) values were 0.204 ± 0.009 for the 2:1 ratio, and 0.274 ± 0.015 for the 3:1 ratio. Regarding their surface charge, both formulations were slightly cationic, as indicated by the obtained zeta potential values of +0.127 ± 0.229 mV and +0.181 ± 0.404 mV for 2:1 and 3:1 ratio, respectively.

### 2.3. Stability in Storage Assessment

The colloidal stability of CS/TPP NPs was evaluated over a storage period of 7 days at 4 °C and solubilized in acetic acid (AcOH) 1%, using both 2:1 and 3:1 ratio. To assess the influence of processing methods on NP stability, three fractions were analyzed: non-centrifuged, supernatant, and NPs following purification with centrifugal concentrators.

**Figure 4:**
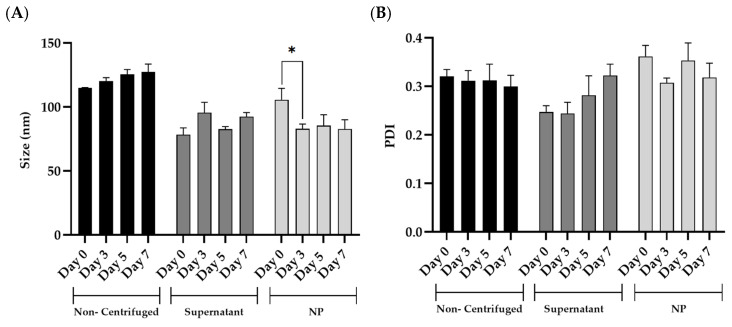
Colloidal stability of CS/TPP NPs at a 2:1 ratio. (**A**) Particle size and (**B**) PDI were measured at 0, 3, 5, and 7 days following NP synthesis. NPs were solubilized in AcOH 1% and stored at 4 °C. Data is presented as mean ± SEM from three independent experiments (n = 3). * *p* < 0.05.

For the 2:1 ratio, non-centrifuged and supernatant samples showed consistent particle size values over 7 days (Figure 4A). For the NP fraction, however, the mean particle size significantly decreased from day 0 to day 3, stabilizing for the remaining days (Figure 4A). All samples for the 2:1 ratio showed consistent PDI values over 7 days (Figure 4B).

**Figure 5:**
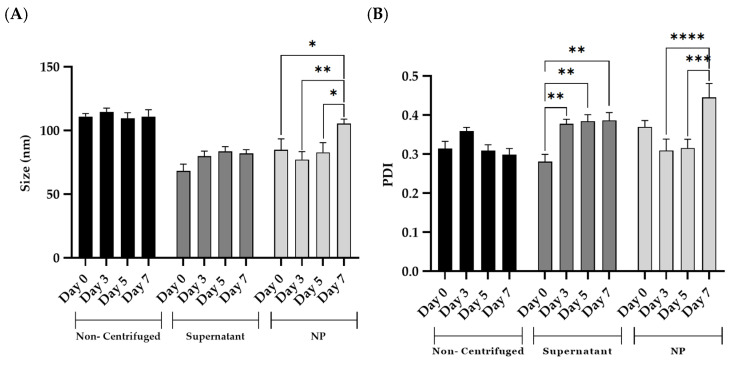
Colloidal stability of CS/TPP NPs at a 3:1 ratio. (**A**) Particle size and (**B**) PDI were measured at 0, 3, 5, and 7 days following NP synthesis. NPs were solubilized in AcOH 1% and stored at 4 °C. Data is presented as mean ± SEM from three independent experiments (n = 3). * *p* < 0.05, ** *p* < 0.01, *** *p* < 0.001, **** *p* < 0.0001.

Additionally, for the 3:1 ratio, measurements indicated that the particle size of NP samples remained statistically unchanged between days 0, 3, and 5. However, on day 7, when compared with earlier time points, a significant increase in particle size was registered (Figure 5A). On the other hand, non-centrifuged and supernatant samples showed consistent particle size values over 7 days (Figure 5A). For supernatant fractions, statistically significant increases in PDI values were registered between day 0 and all of the remaining days. Moreover, for NP samples, PDI values were significantly higher for day 7, when compared with days 3 and 5 (Figure 5B). PDI of non-centrifuged samples remained unchanged over 7 days (Figure 5B).

Given the more adequate combined colloidal properties, the ratio 2:1 was selected for the subsequent in vitro assays.

### 2.4. Cell Viability Assay

**Figure 6:**
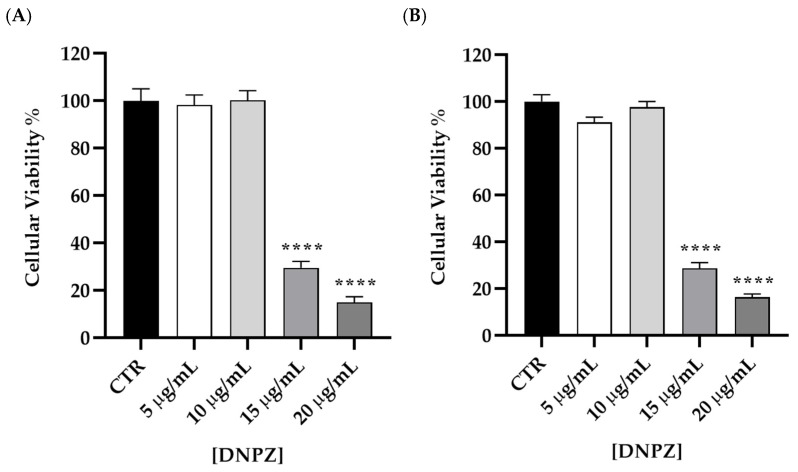
Cellular viability (%) of HIBCPP cells assessed by MTT assay. HIBCPP cells were treated with (**A**) CS/TPP and (**B**) CS/TPP/DNPZ NPs. Data is presented as mean ± SEM of two independent experiments (n = 2). Statistical analysis was performed using one-way ANOVA followed by Dunnett’s test for multiple comparisons. **** *p* < 0.0001.

The biocompatibility of CS/TPP and CS/TPP/DNPZ delivery systems with HIBCPP cells was assessed through the 3-(4,5-dimethylthiazol-2-yl)-2,5-diphenyltetrazolium bromide (MTT) assay. At 15 and 20 µg/mL, blank NPs significantly reduced cellular viability following a 4-h incubation, reaching values of 33.03% ± 4.68 and 14.97% ± 2.28, respectively (Figure 6A). Using the same concentrations, a 4-h incubation with DNPZ-loaded NPs also culminated in a significant decrease in viability. Quantitative analysis revealed that viability decreased to 28.72% ± 4.94 at 15 µg/mL, and 16.35% ± 4.33 at 20 µg/mL (Figure 6B). This result may be due to the presence of a residual amount of AcOH 1% in the final NP suspensions, as this solvent, in a range of 0.01% to 0.5%, has been shown to induce cell death in a dose-dependent manner in normal and neoplastic cell lines of rat’s gastric mucosa [36]. In contrast, exposure to 5 and 10 µg/mL of both blank and DNPZ-loaded CS NPs did not elicit significant reductions in cell viability. As a residual byproduct of the synthesys, AcOH may also contribute to restricting in vivo applicability. However, the obtained nanossystems remain adequate for the assays proposed in the present study.

Based on these results and consistent with similar studies describing the biocompatibility of nano-scale delivery systems at DNPZ concentrations of 10 µg/mL [37], this concentration was selected for subsequent assays.

### 2.5. Circadian Oscillations in DNPZ Transport Across the BSCFB

The transport of DNPZ across the BCSFB was assessed using a transport assay conducted in an in vitro model employing the HIBCPP cell line [38,39]. At the same time, assays were performed to compare the transport profiles of free DNPZ and a mix of CS/TPP/DNPZ NPs and free DNPZ.

**Figure 7:**
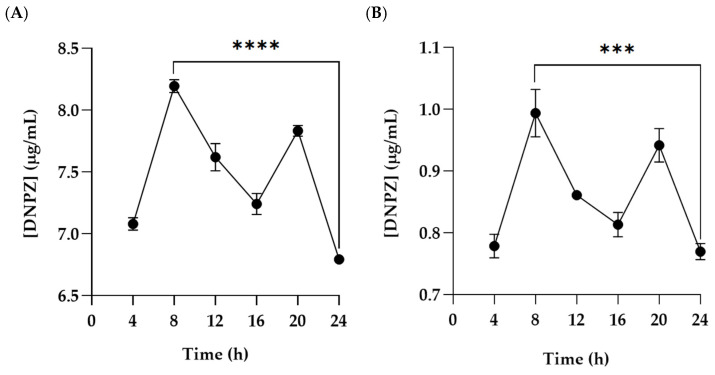

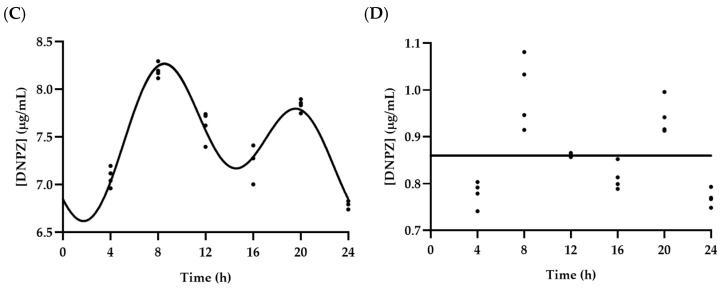
Circadian transport profile of free DNPZ in an in vitro model of the BCSFB. (**A**) Basolateral and (**B**) apical data plotted with GraphPad Prism 8.0.1 and analyzed by one-way ANOVA followed by Tukey’s test for multiple comparisons. Values are presented as mean ± SEM. The difference in the time points of maximum (T8) and minimum (T24) DNPZ concentration within both compartments was statistically significant (*** *p* < 0.001, **** *p* < 0.0001). (**C**) Basolateral and (**D**) apical data graphics following CircWave analysis. Oscillations in DNPZ levels within the basolateral compartment were considered significantly rhythmic (*p* < 0.05) with an associated CoG of 11.40 h. On the other hand, the yielded slope of zero reflects no significant rhythmic variation in the data of the apical compartment (*p* > 0.05).

With respect to the transport of free DNPZ, a one-way ANOVA followed by Tukey’s test for multiple comparisons showed statistical significance between the maximum (T8) and minimum (T24) peaks in DNPZ concentration in both basolateral (Figure 7A) and apical (Figure 7B) compartments. Daily oscillations in DNPZ levels on the basolateral compartment were considered statistically rhythmic when subjected to CircWave analysis (*p* < 0.05) with an associated CoG of 11.40 h (Figure 7C). In contrast, no statistically significant rhythm was detected in the apical compartment following an identical analysis (Figure 7D). Moreover, neither JTK_CYCLE (Table 4) nor Single- and Multi-Component cosinor model analysis yielded significant rhythmicity.

**Figure 8:**
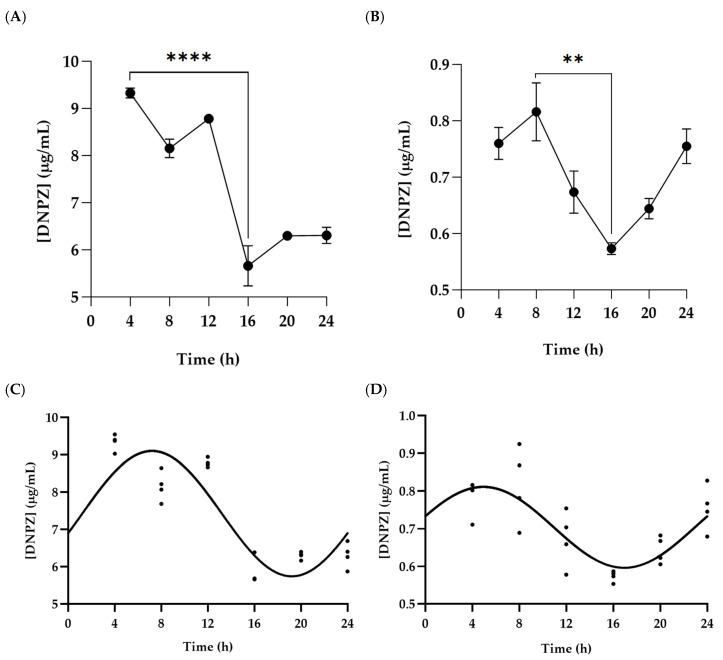
Circadian transport profile of CS/TPP/DNPZ and free DNPZ mix in an in vitro model of the BCSFB. (**A**) Basolateral and (**B**) apical data plotted with GraphPad Prism 8.0.1 and analyzed by one-way ANOVA followed by Tukey’s test for multiple comparisons. Values are presented as mean ± SEM. The difference between the time points of maximum (T4 for basolateral and T8 for apical) and minimum (T16) DNPZ concentration was statistically significant (** *p* < 0.01, **** *p* < 0.0001). (**C**) Basolateral and (**D**) apical data graphics following CircWave analysis. Oscillations in DNPZ levels were considered significantly rhythmic (*p* < 0.05) with CoG values of 7.21 h and 4.94 h in the basolateral and apical compartments, respectively.

Addressing the transport profile of DNPZ-loaded NPs combined with free DNPZ, a one-way ANOVA followed by Tukey’s test for multiple comparisons showed statistical significance between the maximum and minimum peaks in DNPZ concentration in both basolateral (Figure 8A) and apical (Figure 8B) compartments. Daily oscillations in DNPZ levels on both compartments were considered statistically rhythmic when subjected to CircWave analysis (*p* < 0.05) with associated CoG values of 7.21 h and 4.94 h in the basolateral (Figure 8C) and apical (Figure 8D) compartments, respectively. Significant rhythmicity was obtained with JTK_CYCLE analysis for the basolateral (*p* = 0.0011) and apical (*p* = 0.00015) compartments (Table 4). JTK_CYCLE analysis revealed that DNPZ levels in the basolateral compartment oscillated with a calculated period of 24 h, an adjusted phase of 2 h, and an amplitude of 0.71. In the apical compartment, the estimated rhythm-associated parameters were a period of 20 h, an adjusted phase of 4 h, and an amplitude of 0.090. The application of Single- and Multi-Component cosinor models to the basolateral and apical compartment data did not reveal statistically significant rhythmicity.

## 3. Discussion

Circadian rhythms play a critical role in regulating cellular processes that influence drug therapeutic performance, including pharmacokinetic and pharmacodynamic events [40]. Growing interest in chronotherapy reflects the need to align treatments with these biological rhythms, although a deeper understanding of underlying molecular mechanisms remains essential. Given the restrictive role of brain barriers in CNS drug delivery, their circadian regulation is particularly relevant for optimizing therapeutic strategies [34,35]. In parallel, nanotechnology offers promising solutions to overcome key limitations in AD treatment, such as poor brain bioavailability and gastrointestinal side effects associated with DNPZ administration [41,42]. Notably, nanoscale systems, including CS-based NPs, enable improved tissue-specific delivery and enhanced therapeutic efficacy [20,22]. In this context, we propose that the synergistic interplay between chronotherapy and nanotechnology may constitute a novel strategy to enhance DNPZ bioavailability within the CNS.

In the present study, it was shown that the core clock gene *BMAL1* and the efflux membrane transporter *ABCG2* are rhythmically expressed in the HIBCPP cell line. Data were collected over a period of 48 h, improving confidence in the observed oscillations. Under physiological conditions, core clock genes have associated oscillation periods of approximately 24 h. In the present report, the fitted periods for *BMAL1* expression were 27.38 h and 28 h using the CosinorPy and JTK_CYCLE algorithms, respectively. These values align with results obtained for peripheral blood mononuclear cells from healthy individuals, in which period values ranged from 20 to 28 h [43]. From the Multi-Component cosinor analysis, our data showed a CoG value of zero. According to the parameter definition, this result indicates that a peak in *BMAL1* mRNA levels occurred at T0 (i.e., at the moment of synchronization). In contrast, JTK_CYCLE analysis yielded a phase value of 18 h, indicating that *BMAL1* levels peaked at T18. We hypothesize that variations in equivalent rhythmic parameters may be attributed to the distinct computational approaches used by each algorithm. Taking into account the adjusted phase value of 18 h, we can assume that the time point of *BMAL1* peak expression falls within the subjective circadian night. These findings are consistent with previous reports in murine CP and suprachiasmatic nucleus samples [44,45,46] and a human cerebral microvascular endothelial cell line [47]. On the other hand, for the calculated CoG value of 0 h, we can consider that the peak in *BMAL1* mRNA levels may also fall at the beginning of the subjective circadian day. This result is consistent with previous findings by Furtado et al. [36], who reported a CoG of 7.23 h in association with *BMAL1* expression in primary cultures of CP epithelial cells derived from newborn rats. In other murine cerebral tissues, *BMAL1* exhibited similar rhythmic patterns, with peak mRNA expression occurring at T10 in the pineal gland [44] and at T2.5 in the dentate gyrus [46].

Regarding *ABCG2* expression, mRNA levels oscillated with a fitted period of 30.53 h. Considering this fact, the CoG values yield maximum mRNA levels at 17.06 h for Single-Component regression and 16.30 h for Multi-Component regression. These values are consistent with those obtained in a primary culture of rat CP epithelial cells, where maximum *ABCG2* levels were observed at 19.52 h [34]. Curiously, as described by Furtado et al. [35], CoG values for *ABCG2* expression data were 13.66 h in the CP of non-ovariectomized female rats, and 10.74 h in cultured HIBCPP cells. When compared with our findings, these discrepancies may be explained by the assumption of a fixed 24-h period in CircWave analysis, rather than the data-driven fitted period estimated by the algorithms implemented in CosinorPy. Distinct circadian expression profiles of *ABCG2* have been described in extra-CNS tissues, with peak levels observed at T10 in the kidneys and at T6 in both the liver and small intestine of mice [48].

To address the objectives of the present work, CS/TPP/DNPZ NPs were synthesized according to the protocol developed by Garg et al. [22]. NPs were prepared using the ionotropic gelation method, and their reproducibility was confirmed by the high yields obtained. These ranged from 95.55% to 99.69% across all considered ratios, consistent with previous reports showing a process yield of 94.30% for an optimized formulation [22]. On the other hand, CE values, which ranged from 7.33% to 14.65%, remained significantly lower than other documented values. In the present research, CE was 14.65% for the 2:1 ratio and 10.45% for the 3:1 ratio; in a study by Azalea et al. [49] for the same CS:TPP ratios, the resulting CE values were 60.56% and 69.32%, respectively. The markedly distinct CE values obtained can be attributed to differences in formulation conditions and materials. Despite applying the same method for NP formulation, the authors incorporated Tween80 into their CS, TPP, and DNPZ stock solutions. The presence of the emulsifier likely contributes to the higher CE values through the stabilization of the formulated nanosystems. We agree that the low CE may represent a limitation for translational potential. Nevertheless, the system remains promising because DNPZ is therapeutically active at relatively low doses, while chitosan and TPP are inexpensive and biocompatible materials. Azalea et al. also described particle sizes of 165.8 nm for the 2:1 ratio and 227.0 nm for the 3:1 ratio [49], contrasting with the significantly lower 90.02 nm and 98.47 nm values reached for each ratio, respectively, in the present study. The significant differences in particle size may also be attributed to the use of Tween80 [49]. In an additional report, an optimized 3:1 ratio also yielded significantly bigger NPs, with average particle sizes reaching 180.2 nm [22]. The increases in particle size may be partly explained by the use of CS with distinct molecular weights (MW), since polymers with higher MW tend to produce larger NPs, as documented in Rodolfo et al. [50]. Moreover, Garg et al. [22] described the use of medium MW CS, whose sizes surpass 190 kDa, constituting a major difference with the protocol described in the present report, as the CS used had a fixed MW of 20 kDa. Yielded zeta potential values were also markedly different from those previously described in the literature. While our 2:1 and 3:1 ratios yielded NP surface charge values of +0.127 mV and +0.187 mV, respectively, the zeta potential of CS-based delivery systems at a 3:1 ratio reached +16.6 mV [22]. This significant difference underscores, once again, the impact of formulation parameters and materials on a system’s physicochemical properties. Although higher absolute zeta potential values generally enhance nanoparticle stability, a near-neutral surface charge may be advantageous for intravenous delivery by reducing non-specific interactions with blood components and biological membranes, thereby favoring biodistribution. Thus, the near-neutral zeta potential observed in this study was considered compatible with the intended application of the nanosystem. Nevertheless, the cationic nature of the NPs is preserved across formulations, allowing potential electrostatic interactions with the negatively charged membranes and improving the cellular uptake of the delivery systems. Regarding the physicochemical properties of the nanosystems, our results suggest the need for protocol optimization, potentially resulting in higher CE values. To achieve this, we suggest using distinct experimental conditions and reagents. For that end, a different crosslinking agent could be employed to modify the properties of the nanosystems, as described, for example, by Abdelgawad et al. [51], as enhanced loading capacity, larger particle sizes, and higher zeta potential values were reported for CS-based NPs in which hexametaphosphate replaced TPP as a crosslinker. In addition to the discrepancies with previous reports, it is important to acknowledge the limitations associated with the physicochemical characteristics of our formulations and their potential implications for future in vivo applications. In particular, low encapsulation efficiency may necessitate the administration of higher or more frequent doses to achieve the desired therapeutic effect.

Considering the transport of free DNPZ, a significant rhythm was detected only in the basolateral compartment, with an associated CoG of 11.40 h. This peak in DNPZ concentration occurs 5 to 6 h before the *ABCG2* mRNA peak. Thus, our findings suggest a potential rhythmic coordination between drug uptake and transporter-mediated efflux. The role of ABCG2 as a membrane transporter whose activity greatly influences the bioavailability of multiple drugs has been described for diverse tissues, including the brain [52] and gastrointestinal tract [53,54]. Unfortunately, our report lacks the data needed to precisely characterize ABCG2-mediated rhythmic DNPZ transport across the BCSFB, as DNPZ is a substrate for other transporters. Considering a uniform monolayer, when administering the drug to either compartment, DNPZ diffusion is highly dependent on transcellular passage. Thus, a complete understanding of the circadian transport profile of DNPZ requires consideration of membrane influx systems governing its uptake and reuptake, which may also be regulated in a circadian manner. Attending to previous reports of daily rhythmic patterns associated with the expression of several SLC-type transporters in the CP of rats [35,55], circadian mechanisms might also regulate the expression or activity of DNPZ-carrying SLC-type transporters. In fact, regulatory circadian mechanisms also influence efflux transport, as evidenced by daily oscillations in ABCB1 function [47,56], a well-characterized carrier of DNPZ, in the brain barriers of murine models. The presence of ABCB1 at the apical membrane of the BCSFB, sharing its localization with ABCG2, together with the reported rhythmic patterns of efflux activity, may have contributed to the observed daily fluctuations in DNPZ levels in the apical compartment. Based on the currently available evidence, we suggest that the existence of simultaneous mechanisms for the influx and efflux of DNPZ, associated with potentially distinct circadian profiles, may have influenced the patterns observed in free DNPZ levels. However, these transporters are discussed as potential contributors to the observed time-dependent transport of DNPZ rather than as confirmed underlying mechanisms. Further validation is required to clarify their involvement and to determine the potential contribution of additional transporters to DNPZ transport across the barrier. An identical experimental design was employed to evaluate the transport of DNPZ when incorporated as a mix of free and encapsulated drug forms. In contrast to the results discussed above, the concentrations of DNPZ within both compartments revealed rhythmic patterns. This change in transport dynamics is likely due to the presence of CS/TPP/DNPZ NPs, as their diffusion across cellular membranes, mainly via endocytosis, constitutes an alternative transport pathway [57]. Expression and function of membrane transporters remain, however, as crucial factors when evaluating the obtained results due to the simultaneous presence of non-encapsulated DNPZ. CoG and adjusted phase values for oscillations in basolateral DNPZ concentrations were 7.21 h and 2 h, respectively. Both values represent time points of maximum DNPZ levels. Still, they differ markedly due to the nature of the data analyzed, as each algorithm is optimized for distinct characteristics in each dataset. On the other hand, for oscillations in apical drug concentration, the CoG was 4.94 h, and the value for the adjusted phase was 4 h. Upon a first analysis, these results seem counterintuitive, as maximum *ABCG2* mRNA levels are only reached 12 to 13 h later, at around T16/T17. Taking into account a temporal lag of approximately 4–6 h between maximal mRNA and protein expression, attributable to post-transcriptional regulatory mechanisms, transporter protein levels are expected to peak during the subjective circadian night (T20–T23), thereby exacerbating the discrepancy with the timing of maximal DNPZ levels observed in the apical compartment.

The presence of additional transport systems with distinct rhythmic expression may have contributed to the identified discrepancies. Once in the cytoplasm, DNPZ may reach the apical compartment via alternative efflux mechanisms, such as ABCB1-mediated transport. Upon reaching the apical compartment, DNPZ remains a target of reuptake mechanisms. Thus, SLC22A2, an influx transporter expressed at the apical membrane of the choroidal epithelium [58], may have further influenced the DNPZ peaks detected within both compartments. SLC22A2 reuptake-promoting activity likely counteracts ABCG2-mediated DNPZ passage into the apical compartment. Together with the existence of rhythmic expression or activity patterns, as described for transporters of the same family [35,55], we suggest that a simultaneous ABCG2 and transporter-mediated influx of DNPZ at the luminal membrane may have conditioned the rhythmic oscillations in drug levels within the apical chamber. Again, the potential involvement of ABCG2, ABCB1, and SLC transporters is inferred from our experimental findings and supported by the existing literature, rather than being directly demonstrated in the present study.

Variations in DNPZ distribution between apical and basolateral compartments could also be influenced by changes in monolayer permeability in the presence of CS. Indeed, prior reports have demonstrated reduced transepithelial electrical resistance values in in vitro models of the small intestinal epithelium following exposure to CS [59] and CS-based nanosystems [60]. This decrease in transepithelial electrical resistance values and the consequent increase in cell permeability may result from the modulation of tight junction pathways through the redistribution or displacement of key tight junction proteins, such as occludins and claudins. Therefore, the effects of chitosan on paracellular permeability in the HIBCPP cell line should be further investigated to better elucidate the mechanisms underlying chitosan-mediated transport across the barrier. On the other hand, additional pharmacokinetic assays to characterize the drug-release profile of optimal nanoformulations are, in our view, essential for a more detailed analysis of transport assay results. For instance, CS-based NPs previously described in the literature presented controlled drug release profiles, sustaining the drug release for longer periods of time [22,61]. If a similar type of profile is associated with the NPs described in the present study, free DNPZ/encapsulated DNPZ ratios could vary—in a significant, but gradual manner—throughout the considered experimental period. As free and encapsulated DNPZ may be substrates for distinct transport pathways, differentially regulated by circadian mechanisms, we hypothesize that varying levels of both drug forms could influence the observed rhythmic patterns of DNPZ transport across the BCSFB.

Despite presenting promising results, we must not dismiss the simplicity of the model employed in the present study. Indeed, our in vitro cell model cannot fully replicate the systemic, physiological, and pharmacokinetic complexities of the in vivo environment. Nevertheless, static culture models using cell culture inserts are well suited for investigating drug transport mechanisms, assessing drug permeability, and evaluating barrier integrity through measurements of transepithelial electrical resistance. 

In addition, not exploring the several layers that constitute the pharmacokinetic and pharmacodynamic profiles of DNPZ-loaded nanosystems, restricts the regulation of our model to a cell-autonomous circadian machinery, lacking the unavoidable conditioning by the clocks distributed across several other tissues that occurs in complex organisms. Although limited, our data support the proposal of combining the two strategies designed to overcome the challenges imposed on drug delivery by naturally occurring barriers when treating complex disorders, such as AD.

In summary, our findings provide relevant evidence supporting the integration of chronotherapy and nanotechnology as a promising strategy to improve the bioavailability of drugs used in the treatment of AD.

The present report significantly contributes to elucidating the underlying molecular mechanisms that may influence the biological performance of therapeutic compounds and associated specialized delivery systems targeted to enhance their delivery.

## 4. Materials and Methods

### 4.1. Cell Line

All cellular experiments were performed using the HIBCPP cell line, derived from a human malignant CP papilloma from a 29-year-old woman. When in culture, the cells maintain an epithelial arrangement and growth with no contact inhibition [62]. HIBCPP cells express microvilli and proteins composing both tight and adherens junctions [38,62]. Additionally, the cells contain active influx and efflux transport systems, which make them suitable for functional analysis [63].

HIBCPP cells were maintained in Dulbecco’s Modified Eagle Medium/Nutrient Mixture F-12 (DMEM/F-12) culture medium (Sigma-Aldrich, Merck, Darmstadt, Germany) supplemented with 10% (*v*/*v*) fetal bovine serum (FBS), 1% (*v*/*v*) penicillin/streptomycin, and 5 μg/mL insulin (Sigma-Aldrich, Merck, Darmstadt, Germany). Cultures were kept in a humid environment at 37 °C and 5% CO_2_. The culture medium was changed every two days.

### 4.2. HIBCPP Cell Culture for BMAL1 and ABCG2 Circadian Expression

HIBCPP cells were seeded in 24-well culture plates (VWR^®^ International, Radnor, PA, USA) at a density of 1.0 × 10^5^ cells/well. Experiments were conducted one week after seeding. On day one of harvesting, the cells were synchronized with dexamethasone (100 nM; Cayman Chemical, Ann Arbor, MI, USA) for 2 h at 37 °C and 5% CO_2_. After incubation, the culture medium was replaced, and cells were collected every 4 h for 48 h (i.e., at 0, 4, 8, 12, 16, 20, 24, 28, 32, 36, 40, 44, and 48 h after synchronization).

### 4.3. Quantitative Real-Time PCR (qPCR)

Total RNA was extracted from harvested HIBCPP cells using TripleXtractor reagent (GRISP, Porto, Portugal) according to the manufacturer’s instructions. Absorbance measurements at 230, 260, and 280 nm were performed to quantify and characterize the purity of RNA samples, using a NanoDrop^TM^ One/OneC Microvolume UV-Vis Spectrophotometer (Thermo Fisher Scientific, Waltham, MA, USA).

The isolated RNA was used as a template for complementary DNA (cDNA) synthesis, which was performed using NZY M-MuLV Reverse Transcriptase (NZYTech Ltd., Lisboa, Portugal), 10× Reaction Buffer (NZYTech Ltd., Lisboa, Portugal), Random Hexamer mix (NZYTech Ltd., Lisboa, Portugal), and dNTPs NZYMix (NZYTech Ltd., Lisboa, Portugal). All procedures were performed according to the manufacturer’s protocol.

Quantitative real-time PCR (qPCR) was performed to assess the daily rhythmic expression of *BMAL1* and *ABCG2* in HIBCPP cells. Human glyceraldehyde-3-phosphate dehydrogenase (GAPDH) was used as the housekeeping gene. Primer sequences are listed in Table 5. qPCR was performed using Xpert Fast SYBR 2× Mastermix (GRISP, Porto, Portugal). Primer efficiency was tested in stock, 1:2, 1:4, and 1:8 solutions of cDNA. Reactions were carried out using CFX Opus 96 Dx (Bio-Rad, Hercules, CA, USA), according to the following protocol: initial 3-min denaturation at 95 °C followed by 40 cycles of 95 °C for 5 s, 62 °C for 30 s, and 72 °C for 10 s. Validation of transcript amplification was performed through melting curve profile analysis and agarose gel electrophoresis of qPCR products. The relative expression of each selected gene was calculated according to the ΔΔC_T_ method.

### 4.4. Synthesis of Donepezil-Loaded Chitosan Nanoparticles

CS/TPP and CS/TPP/DNPZ NPs were synthesized through the ionotropic gelation method, and the procedure was adapted from a protocol previously established by Garg et al. [22].

Stock ultra-low MW CS (20 kDa) solutions (1 mg/mL) were prepared by dissolving the respective powder in an appropriate volume of AcOH 1%, (pH = 3). Stock TPP solutions (1 mg/mL) were prepared by dissolving the reagent in an appropriate volume of Milli Q water. Both solutions were left under magnetic stirring overnight. The solutions were filtered with a 0.45 μm syringe filter and stored at room temperature. DNPZ (Cayman Chemical, Ann Arbor, MI, USA) stock solution (1 mg/mL) was prepared by resuspending 10 mg of the powder formulation in 10 mL of AcOH 1% under magnetic stirring overnight. The solution was filtered with a 0.45 μm syringe filter and stored at 4 °C until use.

Under constant stirring in a vortex, 100 μL of TPP were added dropwise to previously prepared mixes of CS and AcOH 1% or CS and DNPZ. All ratios considered in the formulation of the systems and corresponding volumes of CS, TPP, AcOH, and DNPZ solutions are presented in Table 6. The systems were left stabilizing at room temperature and, afterwards, subjected to a 30 min centrifugation at 14,000 rpm and 4 °C. Supernatant fractions were treated using Vivaspin^®^6, 100 kDa MWCO polyethersulfone centrifugal concentrators (Merck, Darmstadt, Germany), promoting the separation of NPs from the remaining free drug fraction.

### 4.5. Characterization of CS/TPP/DNPZ Nanoparticles

Empty and loaded systems were characterized by dynamic light scattering using Zetasizer Nano ZS (Malvern Instruments Ltd., Malvern, UK) and the corresponding Malvern Zetasizer Software (v7.13). The evaluated parameters included size, PDI, and zeta potential. Size and PDI measurements were conducted by adding 70 μL of the NP-containing solution to a plastic disposable cell. For particle size determination, a He-Ne laser at 633 nm with non-invasive backscatter was employed. Moreover, to measure the zeta potential, 750 μL of the solution was added to a specialized folded capillary cell. For the surface charge assessment, an electrophoretic light-scattering system using an M3-Phase Analysis Light Scattering laser was considered.

#### Determination of Process Yield and Complexation Efficiency

DNPZ content in formulated nanosystems was assessed through UV-Vis spectrophotometry at a wavelength of 271 nm using VWR^®^ P9 UV/Visible Spectrophotometer (VWR International, Radnor, PA, USA).

For CS/TPP and CS/TPP/DNPZ NPs, the absorbance was measured for non-centrifuged, pellet, supernatant, and fractions from the upper and lower compartments of the centrifugal concentrators. Synthesis-associated yield was assessed using the following formula:

(1) $$\mathrm{Yield}\;(\%)=\frac{\left[\mathrm{DNPZ}\right]\;\mathrm{Supernatant}\;\mathrm{or}\;\left[\mathrm{DNPZ}\right]\;\mathrm{Pellet}}{\left[\mathrm{DNPZ}\right]\;\mathrm{Non}\text{-}\mathrm{Centrifuged}}\times 100$$

CE values were obtained following the separation of free DNPZ from the formulated CS/TPP/DNPZ NPs, and relied on the use of the following formula:

(2) $$\mathrm{CE}\;(\%)=\frac{\left[\mathrm{DNPZ}\right]\;\mathrm{Upper}\;\mathrm{Compartment}\;\mathrm{or}\;\left[\mathrm{DNPZ}\right]\;\mathrm{Lower}\;\mathrm{Compartment}}{\left[\mathrm{DNPZ}\right]\;\mathrm{Supernatant}}\times 100$$

### 4.6. Cellular Viability Assays

Cellular viability assays were conducted using the MTT method. HIBCPP cells were seeded in a 96-well culture plate at a density of 2.0 × 10^4^ cells/well. After 48 h, the culture medium was discarded, and fresh medium with distinct concentrations of CS/TPP or CS/TPP/DNPZ delivery systems was added to the corresponding experimental groups. DNPZ-loaded NPs were tested at 5, 10, 15, and 20 μg/mL of DNPZ, and equivalent dilutions were made for the empty systems. The selection of these concentrations followed previously published studies that employed similar DNPZ dosing ranges for pharmacokinetic and biological performance assessments [34,37]. The negative (CTR) and positive (CTR+) control groups were not incubated with the systems. Following a 4-h incubation, the medium was removed, and the MTT solution (Sigma-Aldrich, Merck, Darmstadt, Germany) was added to each well. CTR+ corresponding wells were incubated with ethanol 70% for 10 min before MTT addition. Cells were incubated at 37 °C for 3 h. The incubation period was followed by the removal of the remaining MTT solution and the addition of dimethyl sulfoxide, which dissolved the formazan crystals. Absorbances were measured at 570 nm in a Bio-Rad xMark Spectrophotometer Microplate Reader (Bio-Rad, Hercules, CA, USA). Viability values are presented as percentages relative to the CTR group.

### 4.7. Free Donepezil and CS/TPP/DNPZ Nanoparticle Transport Assay

As described in the literature, cultured HIBCPP cells express functional transport systems and cellular junction proteins, constituting a valid in vitro model of the BCSFB [63]. HIBCPP cells were cultivated in a standard culture model.

**Figure 9:**
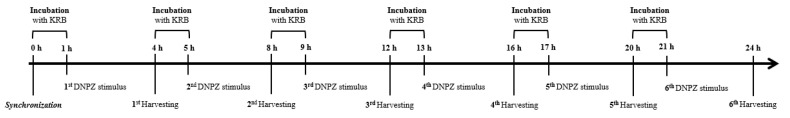
Chronogram of the free DNPZ and free DNPZ + CS/TPP/DNPZ NPs uptake assay. At 0 h, HIBCPP cells were synchronized with dexamethasone. At six distinct time points following synchronization, the cells were incubated with free DNPZ or free DNPZ + CS/TPP/DNPZ NPs mix in the basolateral compartment for 3 h. DNPZ levels in the apical and basolateral compartments were assessed by high-performance liquid chromatography.

**Figure 10:**
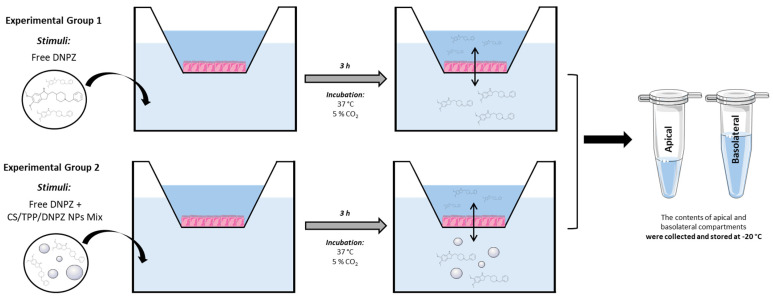
Schematic representation of stimuli administration and harvesting in uptake assays. HIBCPP cells cultivated in a standard culture model were incubated with dexamethasone for a period of 2 h. Afterwards, free DNPZ and free DNPZ + CS/TPP/DNPZ NPs stimuli were administered within the basolateral compartments. Harvesting of apical and basolateral compartments occurred 3 h following stimuli administration. All samples were stored at −20 °C until use. Symbols adapted from Servier Medical Art (https://smart.servier.com).

Cells were seeded in inserts for 24-well cell culture plates (VWR^®^ International, Radnor, PA, USA), with a 0.4 μm pore diameter and a 0.33 cm^2^ insert area, at a density of 1.5 × 10^5^ cells/insert. Cells were maintained in DMEM/F-12 culture medium supplemented with 10% (*v*/*v*) FBS, 1% (*v*/*v*) penicillin/streptomycin, and 5 μg/mL insulin. At day one after seeding, the medium was discarded, and fresh medium was added to the apical compartment. On the second day of culture, the medium was discarded, and fresh volume was added to the apical and basolateral compartments. On the fourth day, the medium in both compartments was replaced with DMEM/F-12 supplemented with 1% (*v*/*v*) FBS, 1% (*v*/*v*) penicillin/streptomycin, and 5 μg/mL insulin. Transepithelial electrical resistance was measured using EVOM^2^ Epithelial Voltohmmeter (World Precision Instruments, Sarasota, FL, USA). On day six following seeding, the culture medium was discarded, fresh medium was added, and transepithelial electrical resistance values were measured. On the seventh day of culture, cells were synchronized through a 2-h incubation with dexamethasone (100 nM) at 37 °C and 5% CO_2_. To ensure adequate exposure, dexamethasone-containing media was administered to both apical and basolateral compartments. Following the synchronization period, fresh medium was added to each insert’s apical and basolateral compartments. To assess potential rhythmic oscillations in the transport of free and encapsulated drug forms across the BCSFB, stimuli were given at six distinct time points (1, 5, 9, 13, 17, and 21 h following synchronization) (Figure 9). Incubations had a duration of 3 h, and free DNPZ or free DNPZ + CS/TPP/DNPZ NP solutions, both at 10 μg/mL of DNPZ, were used. For both experimental groups, four replicates were evaluated per time point (n = 4). Stimuli were administered within the basolateral compartments. All solutions were prepared in Krebs–Ringer Buffer (KRB). Following the incubation period, apical and basolateral contents were collected and stored at −20 °C. A schematic representation of stimuli administration and harvesting is presented in Figure 10.

#### 4.7.1. Donepezil Quantification

**Figure 11:**
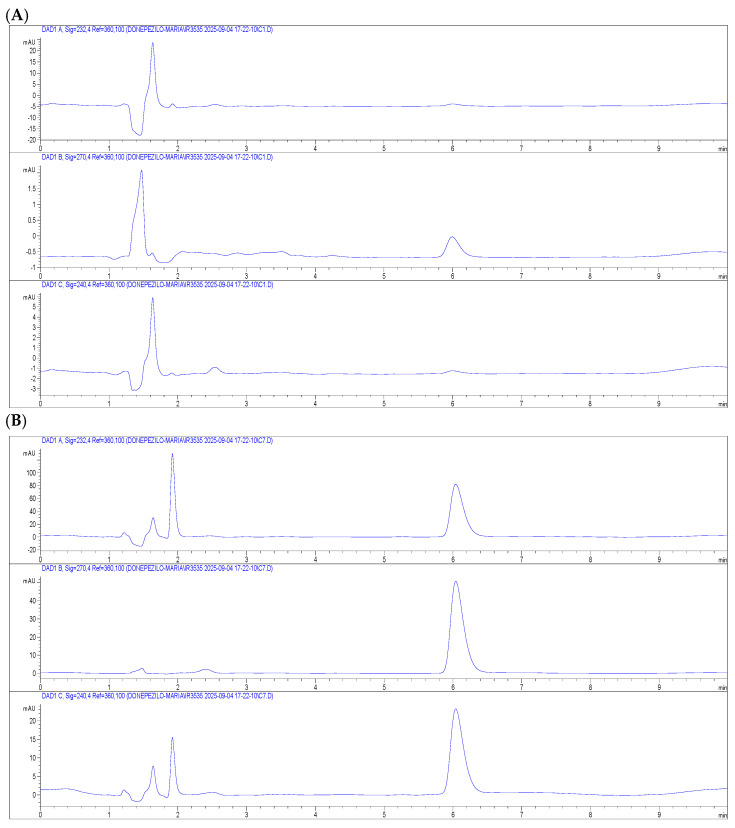
Representative chromatogram obtained for (**A**) DNPZ at 0.16 µg/mL and (**B**) DNPZ at 10 µg/mL at 232, 270, and 240 nm, respectively.

DNPZ was quantified through high-performance liquid chromatography. The chromatographic system was composed of a binary pump model 1290 coupled to a diode-array detector model 1260 from Agilent Technologies (Soquímica, Lisboa, Portugal). Separation was performed in a YMC-Triart PFP analytical column (100 mm × 4.6 mm i.d., 5 μm), protected by a pre-column, from Agilent Technologies (Soquímica, Lisboa, Portugal). The mobile phase consisted of acetonitrile and ammonium formate (5 mM), in a 70:30 (*v*/*v*) ratio, as solvents A and B, respectively. An isocratic elution was carried out at a 1 mL/min flow rate, and the injection volume was 50 μL. The autosampler and column temperatures were set to 25 °C and 40 °C, respectively. The run time for each sample was 15 min, and the average DNPZ retention time was approximately 6 min. DNPZ determination was performed at the following wavelengths: 232, 240, and 270 nm (Figure 11). The selectivity of the HPLC method was assessed by analysing blank nanoparticle formulations and experimental buffer controls (n = 10). No interfering peaks were observed at the retention time of DNPZ, confirming that the nanoparticle components and buffers did not interfere with its chromatographic determination. 

#### 4.7.2. Validation Procedure

The previously described analytical method was validated in accordance with the Food and Drug Administration guidelines [64]. Evaluated parameters included selectivity, linearity, limit of quantification (LLOQ), limit of detection (LOD), precision, and accuracy. The method’s selectivity was analyzed through the assessment of potential interferences (e.g., compounds of KRB), and no other signals were detected at the retention time of DNPZ. Linearity was evaluated in a concentration range from 0.16 μg/mL (LLOQ and LOD) to 10 μg/mL. The criteria for the fitness of the linear model assessment included a weighted determination coefficient (R^2^) greater than 0.99. The linearity data are shown in Table 7, and the values of slope, intercept, and R^2^ are reported as mean ± standard deviation.

Intra- and inter-day precision protocols were adopted to further validate the method, using the following concentrations: 0.16, 0.31, 2.5, and 10 µg/mL. Precision was assessed through the coefficients of variation (CV), for which values equal to or lower than 15% were accepted. Additionally, the method was considered accurate from mean relative error (RE) values of ±20% for the LLOQ and ±15% for the remaining concentrations. Data for intra- and inter-day precision and accuracy are shown in Table 8.

### 4.8. Statistical Analysis

Data sets from stability in storage, and cell viability assays were analyzed for statistical significance between experimental groups through two-way ANOVA, followed by Tukey’s multiple comparisons, and one-way ANOVA, followed by Dunnett’s multiple comparisons, respectively. DNPZ 24-h transport assay data were analyzed through one-way ANOVA, followed by Tukey’s multiple comparisons. Results were considered statistically significant when *p* < 0.05. All data is shown as mean ± standard error of the mean (SEM).

Rhythmicity of *BMAL1* and *ABCG2* expression and DNPZ concentration in the two compartments (apical and basolateral) was assessed using three distinct algorithms: the JTK_CYCLE v3.1 algorithm, integrated in the MetaCycle v1.2.0 package; CosinorPy v2.1 package, and CircWave v1.4 software. Outlier exclusion based on Z-score calculation was executed before the algorithm application. JTK_CYCLE and CosinorPy algorithms were applied to 48 h gene expression data. CircWave v1.4 software allowed only a maximum interval of 24 h.

JTK_CYCLE is a non-parametric method (i.e., data do not require a predefined distribution model) and, therefore, is suitable for analyzing large and robust datasets [65]. For JTK_CYCLE, values obtained for period (JTK_period), *p*-value (JTK_*p*value), adjusted *p*-value (JTK_BH.Q), amplitude (JTK_amplitude), and adjusted phase (JTK_adjphase) were considered in gene expression data analysis. On the other hand, the CosinorPy package includes a parametric method that makes specific assumptions about the data distribution [66]. CosinorPy was used to apply both Single-Component and Multi-Component models. Suitability of the models was assessed through Residual Sum of Squares and Model Sum of Squares value analysis. For CosinorPy, the calculated fitted period, MESOR, amplitude, acrophase, CoG, and *p*-value were considered in gene expression data analysis. The CosinorPy package provided graphical representations alongside the values for rhythmicity parameters. Using the CircWave v1.4 software, datasets were analyzed by a harmonic regression method, with an alpha set at 0.05 and an assumed period of 24 h. CoG and *p*-values were considered in gene expression data analysis. The resulting fitted curve was plotted with GraphPad Prism 8.0.1. Results were considered statistically significant when *p* < 0.05.

## Figures and Tables

**Table 1:** Summary of rhythmicity parameters obtained with JTK_CYCLE for *BMAL1* and *ABCG2* expression.

Gene	JTK_Period (h)	JTK_*p* Value	JTK_Adjusted Phase (h)	JTK_Amplitude
*BMAL1*	28	0.03	18	0.19
*ABCG2*	28	1.00	10	0.13

**Table 2:** Summary of rhythmicity parameters obtained with Single-Component and Multi-Component models from the CosinorPy package.

Gene	Period	*p*	MESOR	Amplitude	Acrophase (Radians)	CoG (h)
Single-Component Model						
*BMAL1*	27.38	0.115	2.22	1.56	0.0	0.0
*ABCG2*	30.53	0.048	1.54	0.56	−2.77	−13.47
Multi-Component Model, adjusted period of 24 h						
*BMAL1*	27.38	0.041	2.22	1.56	0.0	0.0
*ABCG2*	30.53	0.140	1.54	0.56	1.05	5.09
Multi-Component Model, adjusted period of 27 h						
*BMAL1*	27.38	0.107	2.22	1.56	0.0	0.0
*ABCG2*	30.53	-	-	-	-	-

**Table 3:** Process yields and CE values for all CS:TPP ratios considered. For the best ratios, average size, PDI, and zeta potential measurements were also performed. Values are presented as mean ± SD.

CS:TPP Ratio	Yield (%)	CE (%)	Size	PDI	Zeta Potential (mV)
1:2	97.65	8.97	-	-	-
1:1	98.05	7.33	-	-	-
4:3	99.69	10.85	-	-	-
3:2	95.55	12.52	-	-	-
2:1	98.73	14.65	90.02 ± 2.152	0.204 ± 0.009	+0.127 ± 0.229
3:1	97.42	10.45	98.47 ± 2.904	0.274 ± 0.015	+0.181 ± 0.404

**Table 4:** Summary of rhythmicity parameters obtained with JTK_CYCLE for free DNPZ, and a mix of CS/TPP/DNPZ NPs and free DNPZ transport across an in vitro model of the BCSFB.

Compartment	JTK_Period (h)	JTK_*p* Value	JTK_Adjusted Phase (h)	JTK_Amplitude
Free DNPZ				
Basolateral	24	0.16	14.00	0.31
Apical	24	0.89	12.00	0.040
CS/TPP/DNPZ NPs and free DNPZ mix				
Basolateral	24	0.0011	2.00	0.71
Apical	20	0.00015	4.00	0.090

**Table 5:** Primer sequences used in BMAL1 and ABCG2 relative expression evaluation through quantitative real-time PCR.

Gene	Primer Sequence	Amplicon Size (bp)
*GAPDH*	Fw: 5′ATG GGG AAG GTG AAG GTC G 3′ Rv: 5′GGG GTC ATT GAT GGC AAC AAT A 3′	108
*BMAL1*	Fw: 5′TCC ACT GAC TAC CAA GAA AGC 3′ Rv: 5′CTG TTC ATT TTA TCC CGA CGC 3′	143
*ABCG2*	Fw: 5′ACG AAC GGA TTA ACA GGG TCA 3′ Rv: 5′CTC CAG ACA CAC CAC GGA T 3′	93

**Table 6:** Preparation conditions of CS/TPP and CS/TPP/DNPZ nanoparticles.

CS:TPP Ratio	CS (μL)	AcOH (μL)	TPP (μL)	AcOH or DNPZ (μL)
1:2	50	250	100	100
1:1	100	200	100	100
4:3	133	167	100	100
3:2	150	150	100	100
2:1	200	100	100	100
3:1	300	0	100	100

**Table 7:** Linearity data (n = 6).

Linear Range (µg/mL)	Linearity			LLOQ (µg/mL)	LOD (µg/mL)
	Slope	Intercept	R^2^		
0.16–10	63.760 ± 2.688	−2.219 ± 2.536	0.9998 ± 8.165 × 10^−5^	0.16	0.16

**Table 8:** Intra- (n = 5) and inter-day (n = 6) precision and accuracy.

Concentration (µg/mL)	Intra-Day Analysis			Inter-Day Analysis		
	Measured	CV (%)	RE	Measured	CV (%)	RE
0.16	0.17 ± 0.02	13.92	−7.73	0.17 ± 0.02	13.50	−10.37
0.31	0.32 ± 0.02	7.53	−1.91	0.32 ± 0.03	8.09	−3.39
2.5	2.48 ± 0.04	1.81	0.82	2.49 ± 0.04	1.78	0.52
10	10.01 ± 0.03	0.34	−0.06	10.01 ± 0.04	0.35	−0.09

## Data Availability

Data is available upon request.

## Abbreviations

The following abbreviations are used in this manuscript:

ABC	ATP-binding cassette
AcOH	Acetic acid
AD	Alzheimer’s disease
BCSFB	Blood–cerebrospinal fluid barrier
cDNA	Complementary DNA
CE	Complexation efficiency
CNS	Central nervous system
CoG	Center of gravity
CP	Choroid plexus
CS	Chitosan
CSF	Cerebrospinal fluid
CTR	Negative control
CTR+	Positive control
CV	Coefficients of variation
DMEM-F12	Dulbecco’s Modified Eagle Medium/Nutrient Mixture F-12
DNPZ	Donepezil
FBS	Fetal bovine serum
GAPDH	Glyceraldehyde-3-phosphate dehydrogenase
HIBCPP	Immortalized human epithelial CP cells
KRB	Krebs–Ringer buffer
LLOQ	Limit of quantification
LOD	Limit of detection
MESOR	Midline estimating statistic of rhythm
MTT	3-(4,5-dimethylthiazol-2-yl)-2,5-diphenyltetrazolium bromide
MW	Molecular weight
NPs	Nanoparticles
PDI	Polydispersity index
qPCR	Quantitative real-time PCR
RE	Relative error
SEM	Standard error of the mean
SLC	Solute carrier
TPP	Tripolyphosphate
